# Synthesis and Photocontrolled Supramolecular Self-Assembly of Azobenzene-Functionalized Perylene Bisimide Derivatives

**DOI:** 10.3390/polym11071143

**Published:** 2019-07-03

**Authors:** Weikang Ling, Xiaoxiao Cheng, Tengfei Miao, Shuangshuang Zhang, Wei Zhang, Xiulin Zhu

**Affiliations:** 1 Suzhou Key Laboratory of Macromolecular Design and Precision Synthesis, Jiangsu Key Laboratory of Advanced Functional Polymer Design and Application, State and Local Joint Engineering Laboratory for Novel Functional Polymeric Materials, College of Chemistry, Chemical Engineering and Materials Science, Soochow University, Suzhou 215123, China; 2 Global Institute of Software Technology, No 5. Qingshan Road, Suzhou National Hi-Tech District, Suzhou 215163, China

**Keywords:** perylene bisimide, azobenzene, supramolecular assembly, photoisomerization, morphology control

## Abstract

Azobenzene (Azo) units were successfully introduced into perylene bisimide (PBI) structures in order to realize the photocontrolling of the morphology of the supramolecular assembly of PBI by a photoisomerization process. A total of three Azo-functionalized perylene bisimide derivatives (**PBI1**, **PBI2**, and **PBI3**) with different alkyl chain lengths were designed and synthesized by imidization of 3,4,9,10-perylene tetracarboxylic dianhydride with the corresponding amines. The structures of these compounds were characterized by proton nuclear magnetic resonance (^1^H NMR) and matrix-assisted laser desorption ionization time-of-flight mass spectrometry (MALDI-TOF-MS). The photoisomerization behaviors of Azo units in PBIs were investigated using ultraviolet-visible (UV-VIS) absorption spectroscopy, which were obviously effected by solvents and the alkyl chain length. Furthermore, the photoisomerization of Azo units has the obviously regulatory effect on the morphology of supramolecular assembly of PBIs, especially for the medium-length alkyl chain-linked Azo-functionalized PBI derivative (**PBI2**). This research realized the photocontrolling of the morphology of the supramolecular assembly of PBI derivatives by photoisomerization of Azo units.

## 1. Introduction

Perylene tetracarboxylic acid bisimides (PBI) have attracted considerable attention on account of their luminescence [1,2,3,4,5,6], n-type semiconductor [7,8,9,10,11] properties in the past few decades, and have found a use as applications in sensors [12], lasers [13], drug release [14], organic electronic devices [15], supramolecular self-assemblies [16], and so on. The strong π-π stacking interactions between the PBI core composed of five condensed benzene rings endow with the formation of supramolecular structures by the intermolecular self-assembly process [17,18,19]. Furthermore, introduction of amide structure to the PBI core provides hydrogen bonding interaction between PBI building blocks. The cooperation of π-π stacking, hydrogen bonding, hydrophobic/hydrophilic interactions, and the van der Waals force drives the formation of the PBI-based supramolecular nanostructures [20,21,22,23]. Recently, much more efforts have been done on the controlling of the local π-stacking arrangements of PBI units, which is typically called “face-to-face” H-aggregates and “head-to-tail” J-aggregates. H-aggregates and J-aggregates show different electroptical properties in which H-aggregates commonly present low fluorescence emission and high n-type carrier mobility, but J-aggregates demonstrate highly fluorescence emission along with obvious red-shift absorption [24]. Significant progress has been achieved for the transformation of H- and J-aggregates. Würther and co-workers first reported the formation of J-aggregates by supramolecular design principles [25]. Furthermore, they presented the successful transformation from H- to J-aggregates of PBI driving by hydrogen-bond directed complexation between melamine and cyanurate units [26]. More recently, Würther et al. designed one-dimensional brickwork J-aggregates of PBI with thermotropic liquid-crystalline [27]. The formation of J-aggregates of PBI can also be produced by other strategies, such as the addition of the inducer [28], introduction of steric alkyl substituents [29], and so on. Meanwhile, the PBI–based helical nanofibers can be produced by a supramolecular assembly of chiral PBI derivative or an achiral one in chiral solvent [30]. The different types of aggregates result in a different morphology of supramolecular nanostructures, demonstrating different electro-optical properties.

Azobenzene (Azo) is one of the classical photoresponsive compounds, the drastic changes of which both in the structure and properties can be caused by the reversible *trans*-*cis* isomerization process. This special property of Azo has been used to regulate the morphology of aggregates [31,32], supramolecular chirality [33,34,35], and gelation property [36,37]. Ajyaghosh et al. reported an Azo-functionalized phenyleneethynylene derivative, the aggregate morphology of which changed from the initially formed nanodots to supramolecular rods by *trans*-*cis* photoisomerization of the Azo unit [38]. Yagai and co-workers reported the supramolecular assembly of a chiral Azo dimer, whose morphology and chirality can be controlled by photoisomerization of Azo moiety [39]. Our group reported the morphology and chirality control of supramolecular structure of achiral Azo-containing polymers in the chiral solvent by reversible *trans*-*cis* isomerization of Azo chromophore [40]. Nevertheless, introducing Azo units into PBI structure and investigating the effect of the *trans*-*cis* photoisomerization process on the morphology of PBI aggregates is still rare so far. Klaus et al. designed a rigid polyphenylene dendrimer containing Azo, pyridyl, and ethynyl entities, which controlled over molecular size, intramolecular energy transfer between Azo and PBI by reversible photoisomerization of Azo units [41]. Tao and co-workers introduced the Azo groups into the bay-position of the PBI, and they found the morphological evolution of the nanostructures from ribbons to spheres driven by the *trans*-*cis* isomerization of Azo units with 365 nm wavelength light irradiation [42].

Herein, we designed and synthesized a series of symmetrical Azo-functionalized PBI derivatives with Azo groups at both sides of a perylene ring. The supramolecular nanostructures were formed driven by the cooperation of π-π stacking of perylene and Azo groups, hydrogen bonding interaction and the van der Waals force. The effects of different alkyl chain lengths on the Azo photoisomerization behavior and the further influence on the PBI nanostructures were also investigated.

## 2. Experiment

### 2.1. Materials

*p*-Anisidine (99%, Macklin, Shanghai, China), phenol (99%, Enox, Jiangsu, China), sodium nitrite (99%, Enox, Jiangsu, China), 1,6-dibromohexane (98%, Adamas, Shanghai, China), methyl gallate (99%, Sinopharm Chemical Reagent Co., Ltd., Shanghai, China), ethylenediamine (99%, Sinopharm Chemical Reagent Co., Ltd., Shanghai, China), 3,4,9,10-perylenetetracarboxylic dianhydride (98%, D&B, Shanghai, China), zinc acetate (99%, Sinopharm Chemical Reagent Co., Ltd., Shanghai, China), imidazole (99%, Sinopharm Chemical Reagent Co., Ltd., Shanghai, China), 4-acetamino phenol (98%, TCI, Shanghai, China), 1-bromohexane (99%, Macklin, Shanghai, China), 1-bromododecane (98%, Macklin, Shanghai, China), and thionylchloride (99%, Sinopharm Chemical Reagent Co., Ltd., Shanghai, China) were used as received. All solvents and other chemical reagents were purchased from Shanghai Chemical Reagent Co., Ltd. (Shanghai, China) and used as received without any further treatment.

### 2.2. Synthesis of PBI1, PBI2, and PBI3

**Scheme 1:**
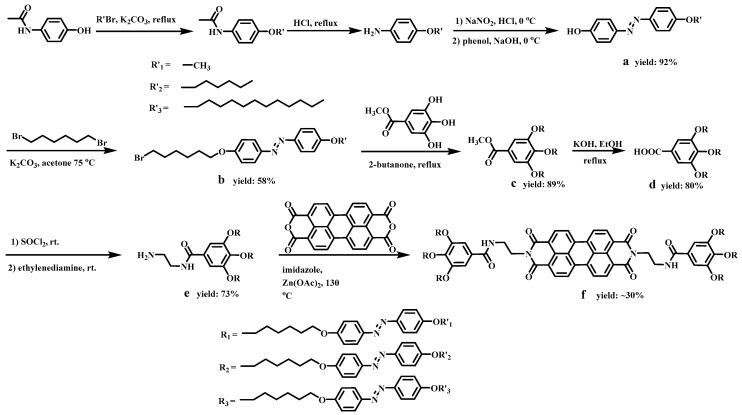
Synthetic routes of perylene tetracarboxylic acid bisimides PBI1, PBI2, and PBI3.

**PBI1**, **PBI2**, and **PBI3** were synthesized according to the procedures in Scheme 1. The compounds a–d were synthesized according to a method similar to that reported by Xie et al. [43] and conformed by proton nuclear magnetic resonance (^1^H NMR) spectra.

**Compound 1a**: Yield 92%. ^1^H NMR (300 MHz, DMSO-*d*_6_) (Figure S1) δ 10.18 (s, 1H, *ArOH*), 7.78 (dd, *J* = 19.5, 8.9 Hz, 4H, *ArH*), 7.01 (dd, *J* = 53.5, 8.9 Hz, 4H, *ArH*), 3.85 (s, 3H, *OCH_3_*). **Compound 2a**: Yield 66%. ^1^H NMR (300 MHz, DMSO-*d*_6_) (Figure S2) δ 10.16 (s, 1H, *ArOH*), 7.77 (dd, *J* = 14.8, 8.9 Hz, 4H, *ArH*), 7.00 (dd, *J* = 47.7, 8.9 Hz, 4H, *ArH*), 4.05 (t, *J* = 6.5 Hz, 2H, *OCH_2_*), 1.88–1.32 (m, 8H, *CH_2_*), 0.88 (t, *J* = 7.0 Hz, 3H, *CH_3_*). **Compound 3a**: Yield 91%. ^1^H NMR (300 MHz, DMSO-*d*_6_) (Figure S3) δ 10.20 (s, 1H, *ArOH*), 7.77 (dd, *J* = 14.4, 8.8 Hz, 4H, *ArH*), 7.00 (dd, *J* = 42.2, 8.9 Hz, 4H, *ArH*), 4.04 (t, *J* = 6.4 Hz, 2H, *OCH_2_*), 1.80–1.13 (m, 22H, *CH_2_CH_2_*), 0.85 (t, *J* = 6.5 Hz, 3H, *CH_3_*).

**Compound 1b**: Yield 58%. ^1^H NMR (300 MHz, CDCl_3_) (Figure S4) δ 8.00–7.84 (m, 4H, *ArH*), 7.00 (dd, *J* = 9.0, 5.0 Hz, 4H, *ArH*), 4.04 (t, *J* = 6.4 Hz, 2H, *OCH_2_*), 3.89 (s, 3H, *OCH_3_*), 3.43 (t, *J* = 6.8 Hz, 2H, *CH_2_Br*), 2.00–1.45 (m, 8H, *CH_2_CH_2_*). **Compound 2b**: Yield 83%. ^1^H NMR (300 MHz, CDCl_3_) (Figure S5) δ 7.88 (d, *J* = 9.0 Hz, 4H, *ArH*), 6.98 (dd, *J* = 9.0, 1.1 Hz, 4H, *ArH*), 4.03 (td, *J* = 6.5, 1.9 Hz, 4H, *OCH_2_*), 3.43 (t, *J* = 6.8 Hz, 2H, *CH_2_Br*), 1.99–1.35 (m, 16H, *CH_2_CH_2_*), 0.92 (t, *J* = 7.0 Hz, 3H, *CH_3_*). **Compound 3b**: Yield 61%. ^1^H NMR (300 MHz, CDCl_3_) (Figure S6) δ 7.97–7.79 (m, 4H, *ArH*), 6.96 (dd, *J* = 11.7, 8.9 Hz, 4H, *ArH*), 4.03 (dd, *J* = 7.9, 5.2 Hz, 3H, *OCH_2_*), 3.42 (q, *J* = 6.6 Hz, 3H, *CH_2_Br*), 2.00–1.13 (m, 30H, *CH_2_CH_2_*), 0.88 (t, *J* = 6.7 Hz, 3H, *CH_3_*).

**Compound 1c**: Yield 89%. ^1^H NMR (300 MHz, CDCl_3_) (Figure S7) δ 7.98–7.73 (m, 12H, *ArH*), 7.27 (s, 2H, *ArH*), 6.96 (ddd, *J* = 10.6, 8.0, 5.0 Hz, 12H, *ArH*), 4.13–3.94 (m, 12H, *OCH_2_*), 3.88 (t, *J* = 4.2 Hz, 12H, *OCH_3_*), 1.97–1.43 (m, 25H, *CH_2_CH_2_*). **Compound 2c**: Yield 83%. ^1^H NMR (300 MHz, CDCl_3_) (Figure S8) δ 7.89 (dd, *J* = 8.9, 1.6 Hz, 12H, *ArH*), 7.27 (s, 2H, *ArH*), 6.97 (dd, *J* = 9.0, 3.1 Hz, 12H, *ArH*), 4.02 (td, *J* = 6.3, 3.4 Hz, 17H, *OCH_2_*), 3.89 (s, 3H, *OCH_3_*), 1.82–1.35 (m, 47H, *CH_2_CH_2_*), 0.91 (t, *J* = 6.8 Hz, 9H, *CH_3_*). **Compound 3c**: Yield 84%. ^1^H NMR (300 MHz, CDCl_3_) (Figure S9) δ 7.99–7.75 (m, 12H, *ArH*), 7.27 (s, 2H, *ArH*), 7.03–6.90 (m, 12H, *ArH*), 4.02 (dd, *J* = 11.3, 6.5 Hz, 17H, *OCH_2_*), 3.89 (s, 3H, *OCH_3_*), 2.01–1.15 (m, 90H, *CH_2_CH_2_*), 0.88 (t, *J* = 6.6 Hz, 9H, *CH_3_*).

**Compound 1d**: Yield 80%. ^1^H NMR (300 MHz, CDCl_3_) (Figure S10) δ 7.84 (ddd, *J* = 12.0, 6.3, 3.6 Hz, 12H, *ArH*), 7.32 (s, 2H, *ArH*), 6.96 (ddd, *J* = 10.5, 7.8, 4.2 Hz, 12H, *ArH*), 4.02 (dq, *J* = 13.3, 6.4 Hz, 12H, *OCH_2_*), 3.86 (s, 9H, *OCH_3_*), 1.96–1.43 (m, 26H, *CH_2_CH_2_*). **Compound 2d**: Yield 85%. ^1^H NMR (300 MHz, CDCl_3_) (Figure S11) δ 7.88 (d, *J* = 8.3 Hz, 12H, *ArH*), 7.33 (s, 2H, *ArH*), 6.96 (d, *J* = 7.5 Hz, 12H, *ArH*), 4.02 (dd, *J* = 13.5, 6.8 Hz, 18H, *OCH_2_*), 2.00–1.23 (m, 48H, *CH_2_CH_2_*), 0.91 (t, *J* = 6.5 Hz, 9H, *CH_3_*). **Compound 3d**: Yield 82%. ^1^H NMR (300 MHz, CDCl_3_) (Figure S12) δ 7.85 (t, *J* = 7.0 Hz, 12H, *ArH*), 7.31 (s, 2H, *ArH*), 6.98 (dd, *J* = 12.0, 4.4 Hz, 12H, *ArH*), 4.14–3.87 (m, 17H, *OCH_2_*), 1.97–1.17 (m, 90H, *CH_2_CH_2_*), 0.88 (t, *J* = 6.5 Hz, 10H, *CH_3_*).

Synthesis of **compounds 1e**, **2e** and **3e** is described further down. **Compound 1d** (2.10 g, 1.9 mmol) was dissolved in dry dichloromethane (30 mL). Thionyl chloride (5 mL) was added along with few drops of dry DMF. The reaction mixture was stirred at room temperature for 12 h. Then, the stirring was stopped and the solvents were evaporated under vacuum. The crude product was obtained as a yellow solid and was taken to the next step without further purification. It was dissolved in dry dichloromethane (30 mL) and the solution was added drop-wise to an ice-cold flask containing ethylene diamine (10 mL). The reaction mixture was stirred in the same ice bath for another 3–4 h and then at room temperature for 12 h. The reaction mixture was diluted with dichloromethane (60 mL) and washed with water, aqueous NaHCO_3_, and brine. The organic layer was dried over Na_2_SO_4_ and the solvent was evaporated to get the crude product, which was then added to ethanol (60 mL) and kept in the refrigerator for 2 h. The precipitate was filtered under vacuum and dried to get the crude product as a yellow solid. It was taken to the next step without further purification. The **compounds 2e** and **3e** were synthesized via the similar procedures. **Compound 1e**: Yield 73%. ^1^H NMR (300 MHz, DMSO-*d*_6_) (Figure S13) δ 8.33 (d, *J* = 7.5 Hz, 1H, *NH_2_*), 7.77 (dd, *J* = 18.0, 10.2 Hz, 12H, *ArH*), 7.16 (s, 2H, *ArH*), 7.13–6.90 (m, 12H, *ArH*), 4.07–3.85 (m, 12H, *OCH_2_*), 3.83 (s, 9H, *OCH_3_*), 3.26 (d, *J* = 6.5 Hz, 2H, *CH_2_*), 2.68 (t, *J* = 6.4 Hz, 2H, *CH_2_*), 1.91–1.30 (m, 25H, *NH,CH_2_CH_2_*). **Compound 2e**: Yield 79%. ^1^H NMR (300 MHz, DMSO-*d*_6_) (Figure S14) δ 8.38 (s, 1H, *NH_2_*), 7.80 (dd, *J* = 13.4, 8.3 Hz, 12H, *ArH*), 7.16 (s, 2H, *ArH*), 7.07 (dd, *J* = 14.1, 8.1 Hz, 12H, *ArH*), 4.00 (dd, *J* = 29.6, 22.4 Hz, 17H, *OCH_2_*), 3.65 (t, *J* = 6.7 Hz, 3H, *CH_2_*), 2.73 (s, 3H, *CH_2_*), 1.74–1.39 (m, 55H, *CH_2_CH_2_*), 0.88 (s, 9H, *CH_3_*). **Compound 3e**: Yield 81%. ^1^H NMR (300 MHz, CDCl_3_) (Figure S15) δ 8.04–7.70 (m, 12H, *ArH*), 6.97 (dd, *J* = 11.5, 4.8 Hz, 12H, *ArH*), 6.62 (s, 2H, *ArH*), 4.11–3.85 (m, 18H, *OCH_2_*), 3.33 (s, 2H, *CH_2_*), 3.05 (s, 3H, *CH_2_*), 1.80–1.41 (m, 92H, *CH_2_CH_2_*), 0.88 (t, *J* = 6.1 Hz, 10H, *CH_3_*).

Synthesis of **compound 1f** (**PBI1**), **2f** (**PBI2**) and **3f** (**PBI3**) is described further down. **Compound 1e** (0.79 g, 0.66 mmol), 3,4,9,10-perylenetetracarboxylic dianhydride (0.13 g, 0.33 mmol), Zn(OAc)_2_ (0.07 g, 0.33 mmol), and imidazole (12.00 g) were taken together in a flask and were stirred at 130 °C for 24 h under an argon atmosphere. The reaction mixture was cooled to room temperature and poured into methanol (100 mL). The precipitate was filtered and the solid was washed several times with methanol. The crude product was recrystallized from THF and dried under vacuum to get 0.26 g of a red solid. **Compounds 2f** (**PBI2**) and **3f** (**PBI3**) were synthesized by the similar procedures. **Compound 1f** (**PBI1**), yield: 30%. ^1^H NMR (300 MHz, DMSO-*d*_6_, Figure S16) δ 8.74 (d, *J* = 7.9 Hz, 4H, *ArH*), 8.47 (d, *J* = 8.0 Hz, 4H, *ArH*), 8.20 (t, *J* = 5.9 Hz, 2H, *NH*), 7.83–7.61 (m, 24H, *ArH*), 7.02–6.90 (m, 28H, *ArH*), 4.29 (t, *J* = 5.5 Hz, 4H, *CH_2_*), 4.01–3.86 (m, 24H, *OCH_2_*), 3.82 (d, *J* = 1.8 Hz, 18H, *OCH_3_*), 3.66 (d, *J* = 7.2 Hz, 4H, *CH_2_*), 1.78–1.35 (m, 48H, *CH_2_CH_2_*). Matrix-assisted laser desorption ionization time-of-flight mass spectrometry (MALDI-TOF-MS), m/z (+Na^+^): calculated for C_156_H_160_N_24_O_16_ (+Na^+^), 2664.18; found, 2664.28. **Compound 2f** (**PBI2**), yield 33%. ^1^H NMR (300 MHz, CDCl_3_, Figure S17) δ 8.55 (d, *J* = 8.0 Hz, 4H, *ArH*), 8.39 (d, *J* = 8.1 Hz, 4H, *ArH*), 7.89–7.66 (m, 24H, *ArH*), 7.02 (s, 4H, *ArH*), 6.99 (s, 2H, *NH*), 6.91 (ddd, *J* = 17.9, 10.7, 6.2 Hz, 25H, *ArH*), 4.51 (s, 4H, *CH_2_*), 4.14–3.90 (m, 36H, *OCH_2_*), 3.87 (s, 5H, *CH_2_*), 1.96–1.29 (m, 140H, *CH_2_CH_2_*), 0.91 (t, *J* = 6.9 Hz, 17H, *CH_3_*). MALDI-TOF-MS, m/z (+Na^+^): calculated for C_186_H_220_N_16_O_24_ (+Na^+^), 3084.30; Found, 3085.71. **Compound 3f** (**PBI3**): Yield 34%. ^1^H NMR (300 MHz, CDCl_3_, Figure S18) δ 8.55 (s, 4H, *ArH*), 8.43 (s, 4H, *ArH*), 7.93–7.63 (m, 24H, *ArH*), 7.02 (s, 4H, *ArH*), 6.88 (d, *J* = 16.6 Hz, 26H, *ArH,NH*), 4.51 (s, 4H, *CH_2_*), 4.19–3.79 (m, 36H, *OCH_2_*), 3.40 (s, 5H, *CH_2_*), 1.79–1.15 (m, 176H, *CH_2_CH_2_*), 0.88 (s, 22H, *CH_3_*). MALDI-TOF-MS, m/z (+Na^+^): calculated for C_222_H_292_N_16_O_24_ (+Na^+^), 3589.21; found, 3590.21.

### 2.3. Preparation of Solutions of PBI Derivatives

Due to the poor solubility of PBI derivatives, we chose DMF (hydrogen bond breaking) and DCE (without hydrogen bond breaking) as solvents for the comparative study. The PBI derivatives were dissolved in DMF and DCE by heating and formed a solution of 1.0 × 10^−5^ mol∙L^−1^, respectively.

### 2.4. Fabrication of Nanostructures

One milliliter of a DMF solution of the compounds **PBI1**, **PBI2**, and **PBI3** (1.0 × 10^−5^ mol∙L^−1^) before and after irradiation with 365 nm and 436 nm wavelength light for 30 min, respectively, was injected into 5 mL of methanol in a test tube followed by 1 h of aging, the assemblies thus formed accordingly. The assemblies can be transferred and casted onto a copper grid by pipetting. It is worth mentioning that the morphological change of the nanostructures in the dark can take place in the solution dispersion, but is restricted in solid state, i.e., when casting on substrates such as a copper grid, the morphologies can remain for more than 3 months.

### 2.5. Characterization

The ^1^H NMR spectra were recorded on a Bruker 300 MHz spectrometer (Bruker, Billerica, MA, USA) and were referenced internally to TMS as an internal standard at 0.00 ppm. Ultraviolet visible (UV-VIS) absorption spectra of the samples were recorded on a Shimadzu UV-2600 spectrophotometer (Kyoto, Japan) at room temperature. Matrix-assisted laser desorption/ionization time-of-flight mass spectrometry (MALDI-TOF-MS) measurement was performed using a Bruker UltrafleXtreme (MALDI-TOF/TOF) mass spectrometer (Bruker, Billerica, MA, USA) equipped with a 355 nm Nd:YAG laser. Both matrix (2-[(2*E*)-3-(4-*tert*-butylphenyl)-2-methyl-prop-2-enylidene] malononitrile (DCTB)) (at a concentration of ca. 20 mg mL^−1^) and all samples were dissolved in chloroform (at a concentration of ca. 10 mg∙mL^−1^). The light intensities of 365 nm and 436 nm were about 2.0 mW cm^−2^ and 0.88 mW cm^−2^, respectively. The light intensity was measured by using a UV radiometer, UV-420 (photoelectric instrument factory of Beijing Normal University). TEM was recorded on HITACHI HT7700 employing an accelerating voltage of 120 kV.

## 3. Results and Discussion

### 3.1. Synthesis and Characterization of Azo-Functionalized PBIs 1–3

**Figure 1:**
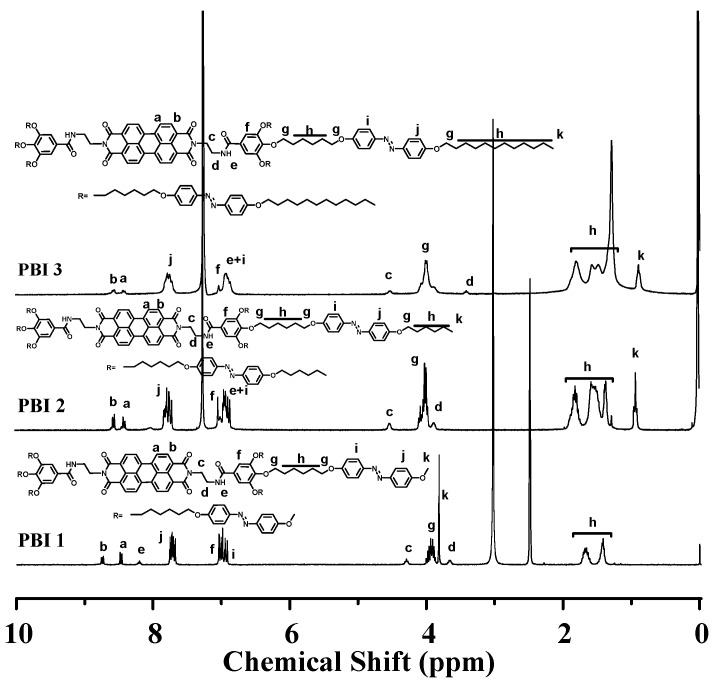
Proton nuclear magnetic resonance (^1^H NMR) spectra of **PBI1** in DMSO-*d*_6_, and **PBI2** and **PBI3** in CDCl_3_.

**Figure 2:**
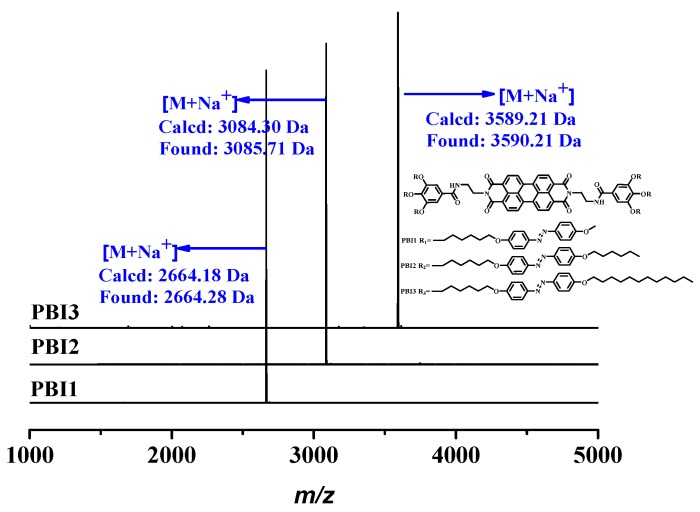
Matrix-assisted laser desorption ionization time-of-flight mass spectrometry (MALDI-TOF-MS) spectra of **PBI1**, **PBI2**, and **PBI3**.

The Azo-functionalized PBI derivatives (**PBIs 1–3**) with different alkyl chain lengths were synthesized by imidization of 3,4,9,10-perylene tetracarboxylic dianhydride with corresponding amines as shown in Scheme 1. The structures of **PBIs 1–3** were characterized by ^1^H NMR and MALDI-TOF-MS spectra. We chose DMSO-*d*_6_ as solvent for **PBI1** and CDCl_3_ for **PBI2** and **PBI3** according to their different solubility for NMR measurement. As shown in Figure 1, there exist multiplet peaks at 8.36–8.78 ppm (perylene-H), multipet peaks at 7.65–7.86 ppm and 6.76–7.06 ppm (Ar-H and Azo-H), multiplet peaks at 3.85–4.15 ppm (–OCH_2_–), 4.25–4.60 ppm (–N–CH_2_–), and 1.10–1.90 ppm (–CH_2_–CH_2_–); triplet peaks at 8.15–8.25 ppm (–CONH–), multiplet peaks at 3.60–3.70 ppm (–NH–CH_2_–), and single peak at 3.80–3.83 ppm (–OCH_3_) of **PBI1**, multiplet peaks at 7.00–7.05 ppm (–CONH–) and 3.35–3.90 ppm (–NH–CH_2_–), triplet peaks at 0.82–0.96 ppm (–CH_2_CH_3_) of **PBI2** and **PBI3** with the ratio of the integrated areas of the peaks in accordance with the proton number of the expected compounds, respectively. Meanwhile, the MALDI-TOF-MS spectra of **PBI1**, **PBI2**, and **PBI3** (Figure 2) demonstrated the corresponding experimental peaks *m*/*z* values with sodium ion at 2664.28, 3085.71, and 3590.21 Da, respectively, which matched well with the theoretical calculating values (2664.18, 3084.30, and 3589.21 Da). All these characteristic data confirmed the successful synthesis of Azo-functionalized PBI derivatives (**PBIs 1–3**).

### 3.2. Photoisomerization Behaviors of PBIs 1–3 in the Solution

**Figure 3:**
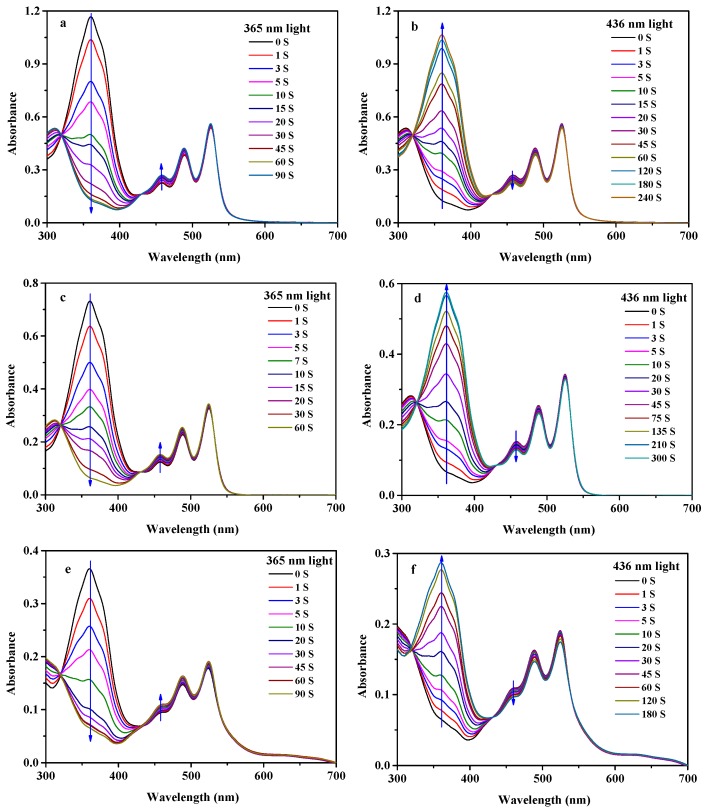
UV-VIS spectra of **PBI1** (**a**,**b**), **PBI2** (**c**,**d**), and **PBI3** (**e**,**f**) assemblies in DMF by alternating irradiation with 365 nm and 436 nm wavelength light.

The synergic interactions of π-π-interaction of perylene and Azo groups, hydrogen bond, and the van der Waals force enforces the PBI derivatives to form supramolecular nanostructures [29]. As shown in Figure 3, the absorption peaks at 360 nm and 458 nm are, respectively, related to the π-π* transition of Azo *trans*-configuration and n-π* transition of Azo *cis*-configuration [39], and the absorption peaks at 490 nm and 520 nm represent absorbance of PBI core [29]. In DMF, the UV-VIS absorption intensities at 360 nm of all the Azo-functionalized PBI derivatives (**PBIs 1–3**) decreased significantly, along with gradual increase of UV-VIS absorption at 458 nm, indicating the *trans*-to-*cis* transformation of Azo units under 365 nm wavelength light irradiation. The *trans*-configuration of Azo groups in PBI structures were almost completely converted to *cis*-configuration and the photostationary state (PPS) time for **PBIs 1–3** were 90 s (**PBI1**), 60 s (**PBI2**), and 90 s (**PBI3**), respectively. Subsequently, the *cis*-to-*trans* transformation of Azo units in PBIs was triggered by irradiating with 436 nm wavelength light. As presented in Figure 3b,d,f, the absorption values at 360 nm (π-π* transition of *trans*-Azo) of **PBIs 1–3** all significantly increased along with the gradual decrease of absorption intensities at 458 nm (n-π* transition of *cis*-Azo), indicating the successful *cis*-to-*trans* photoisomerization. The PPS times for **PBIs 1–3** were 240 s, 300 s, and 180 s, respectively.

**Figure 4:**
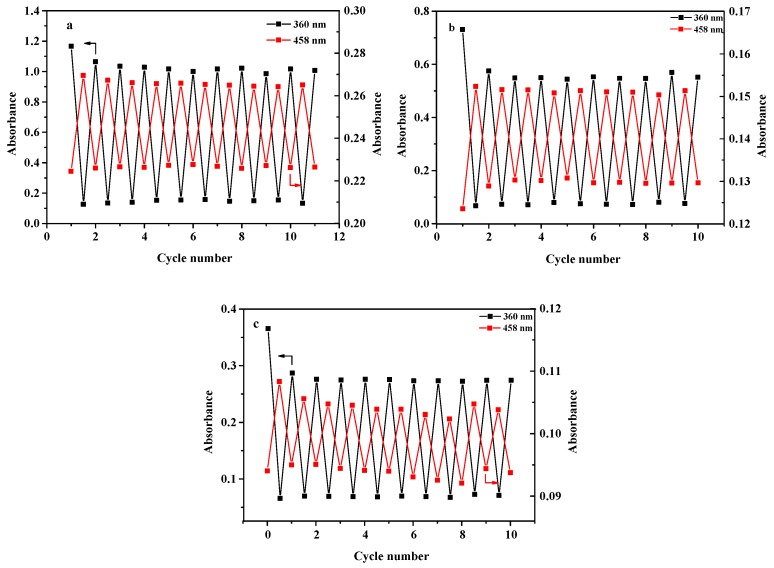
Reversible photoisomerization of **PBI1** (**a**), **PBI2** (**b**), and **PBI3** (**c**) aggregates in DMF upon alternating irradiation with 365 nm and 436 nm wavelength light at 25 °C. The irradiation time: 3 and 5 min for 365 nm and 436 nm wavelength light. The absorption value change for *trans*- and *cis*-configuration was taken from 360 nm (black) and 458 nm (red), respectively.

The reversibility of the photoisomerization of **PBIs 1–3** was verified by alternating irradiation with 365 nm and 436 nm wavelength light for 3 min and 5 min, respectively. As seen in Figure 4, the reversible changes of UV-VIS absorption intensities were tested in cases of **PBIs 1–3**, which could proceed more than ten times. Meanwhile, almost no obvious difference of photoisomerization behavior was detected among **PBIs 1–3** with different alkyl chain lengths in Azo units. The good fatigue resistance and optical switch properties provided us a basis for the subsequent morphology regulation of PBI aggregates.

**Figure 5:**
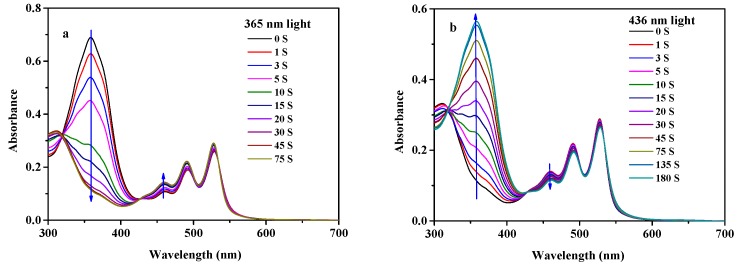

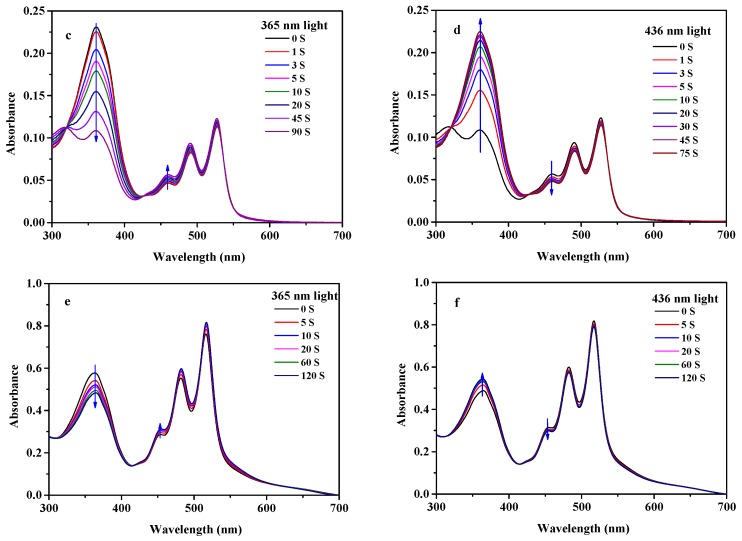
UV-Vis spectra of **PBI1** (**a**,**b**), **PBI2** (**c**,**d**), and **PBI3** (**e**,**f**) assemblies in DCE by alternating irradiation with 365 nm and 436 nm wavelength light.

**Figure 6:**
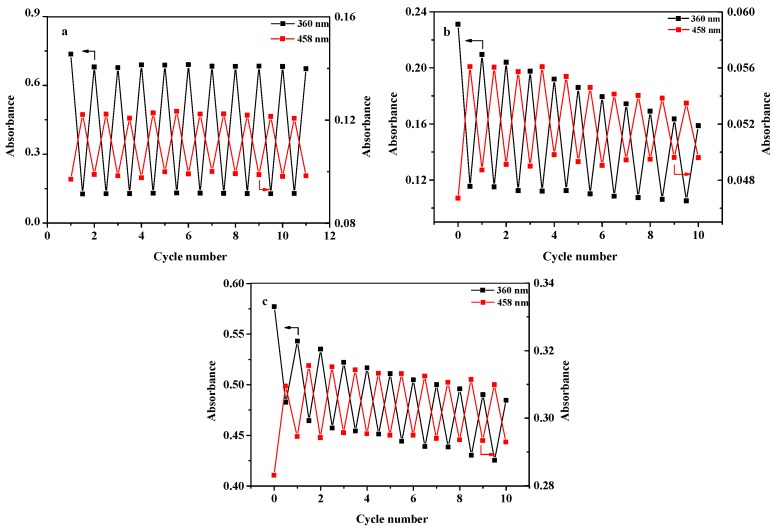
Reversible photoisomerization of **PBI1** (**a**), **PBI2** (**b**), and **PBI3** (**c**) aggregates in DCE upon alternating irradiation with 365 nm and 436 nm wavelength light at 25 °C. The irradiation time: 3 and 5 min for 365 nm and 436 nm wavelength light. The absorption value change for *trans*- and *cis*-configuration was taken from 360 nm (black) and 458 nm (red), respectively.

Considering the different hydrogen bond (N–H···O=C) ability between PBI and the solvent, the similar photoisomerization behaviors of **PBIs 1–3** were investigated in DCE. As shown in Figure 5, the change tendency of the absorption intensities at 360 nm and 458 nm positions were similar to those in DMF. Interestingly, it was notable that the photoisomerization ability of **PBIs 1–3** became weaker with the increase of alkyl chain length. It means that the photoisomerization ability followed the order of **PBI1** > **PBI2** > **PBI3.** The possible reason may be that the longer the alkyl chain, the stronger van der Waals force between PBI units, which impedes the photoisomerization of Azo units. By comparing the UV-VIS absorption spectra of **PBIs 1–3** in DMF and DCE, the photoisomerization ability of **PBIs 1–3** in DCE was much weaker than that in DMF, especially for **PBI2** and **PB 3**. The reason is that the hydrogen bond between DMF and PBI depresses the hydrogen bond interaction between PBI units [29]. Furthermore, the reversibility of the photoisomerization of **PBIs 1–3** also became weaker with the increase of alkyl chain length as demonstrated in Figure 6. In the cases of **PBI2** and **PBI3**, some aggregates precipitated from the solvent after several photoisomerization cycles due to the much stronger interactions between PBI units, which resulted from the increase of van der Waals force with the longer alkyl chain length.

The concentration- and temperature-dependent UV-VIS spectra of **PBIs 1–3** were further investigated and results are given in Figures S19 and S20. Figure S19 shows, the UV-VIS absorption intensities for Azo and PBI unit in **PBIs1–3** all enhanced with the increase of concentration in both DMF and DCE. Interestingly, the relationship of UV-VIS absorption intensity and concentration is not linear (inserted images) for **PBIs1–3**, demonstrating there were aggregates that existed. Figure S20 shows that only UV-Vis absorption intensity of **PBI3** in the DMF solution increased with temperature (from 30 to 90 °C); however, the UV-VIS absorption intensities of DMF solutions of **PBI1** and **PBI2** and DCE solutions of **PBIs 1–3** all increased with the decrease of temperature. Meanwhile, the slight red-shift of absorption peaks with a decrease in temperature were observed in the cases of DMF solutions of **PBI1** and **PBI2** and DCE solutions of **PBIs 1–3**. This phenomenon is unusual and the reason is indistinct, which is why it needs further study in the future.

**Figure 7:**
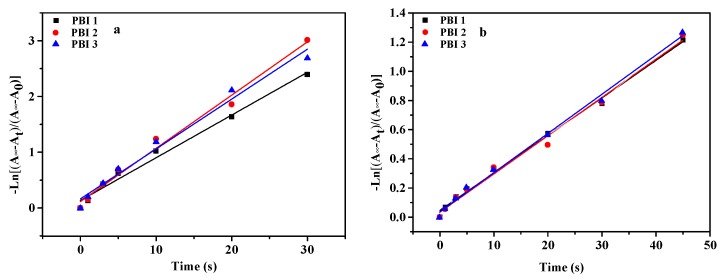
First-order kinetics for the photoisomerization of **PBIs 1–3** by irradiation with 365 nm (**a**) and 436 nm (**b**) wavelength light in DMF.

**Figure 8:**
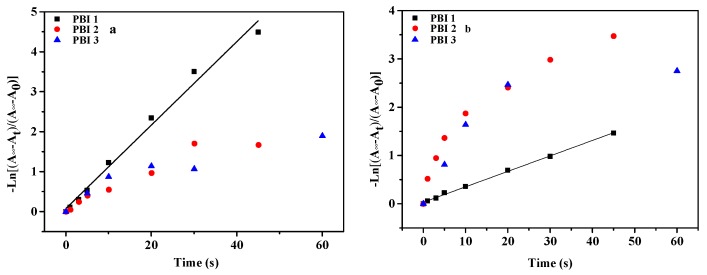
First-order kinetics for the photoisomerization of **PBIs 1–3** by irradiation with 365 nm (**a**) and 436 nm (**b**) wavelength light in DCE.

To further investigate the difference of photoisomerization rates of **PBIs 1–3** in DMF and DCE, we calculated the photoisomeriaztion rate constants of *trans*-to-*cis* (*K*_e_) and *cis*-to-*trans* (*K*_h_) of the Azo units. The *K*_e_ and *K*_h_ values are calculated by fitting the experimental data to the following equation [44,45]:

ln[(A_∞_ − A_0_)/(A_∞_ − A_t_)] = *K*t (1)

where A_∞_, A_0_, and A_t_ are absorbances at 360 nm at infinite time, time zero, and time t, respectively. Figure 7 indicates the first-order kinetic plots of the *trans*-to-*cis* and *cis*-to-*trans* photoisomerization of the **PBIs 1–3** in DMF. The corresponding *K*_e_ and *K*_h_ values are listed in Table 1. Obviously, their *trans-cis* photoisomerization behavior in DMF obeyed the first-order reaction. Under 365 nm wavelength light irradiation, the *K*_e_ values of **PBI1**, **PBI2**, and **PBI3** are 0.0767 s^−1^, 0.0955 s^−1^, and 0.0896 s^−1^, respectively. The photoisomerization rate of **PBI2** is slightly faster, but the difference with that of **PBI1** and **PBI3** was small. Under 436 nm wavelength light irradiation, the *K*_h_ values of **PBI1**, **PBI2**, and **PBI3** are 0.0257 s^−1^, 0.0262 s^−1^, and 0.0269 s^−1^, respectively, which show almost no difference. In DCE, as shown in Figure 8, only **PBI1** showed the first-order kinetic photoisomerization process. The *K*_e_ value is 0.1045 s^−1^ and the *K*_h_ value is 0.0320 s^−1^, which is close to that in DMF.

### 3.3. Morphology Photocontrol of PBIs 1–3 in DMF

**Figure 9:**
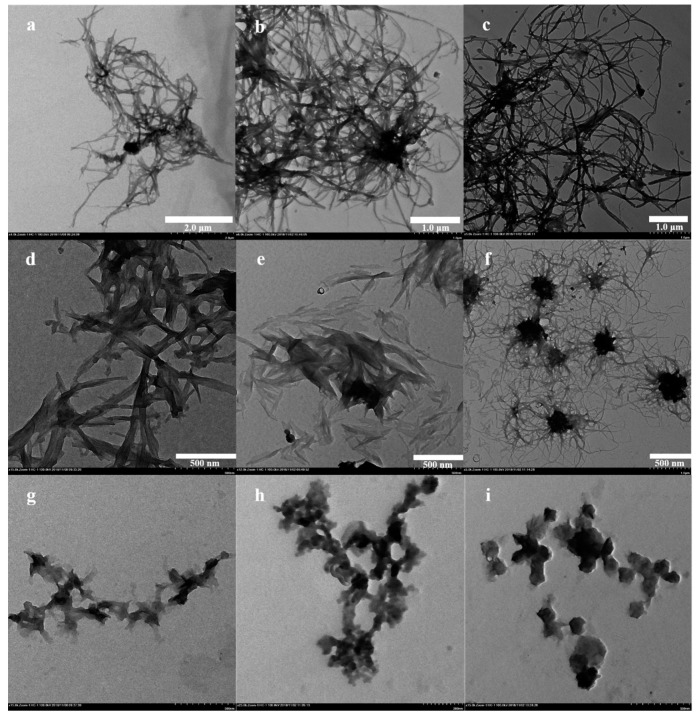
TEM images of **PBI1** (**a**–**c**), **PBI2** (**d**–**f**), and **PBI3** (**g**–**i**) aggregates in DMF/CH_3_OH. From left to right: Before irradiation, irradiated with 365 nm wavelength light for 30 min, irradiated with 436 nm wavelength light for 30 min, respectively.

As compared in the photoisomerization behaviors in different solvents, **PBIs 1–3** took the reversible photoisomerization in DMF, which provided a clue for the morphology control of the PBIs aggregates. Figure 9 presents the typical TEM images of **PBIs 1–3** assemblies in DMF. **PBI1** formed the well-defined long fibers with the width of 90 ± 2 nm and several micrometers in length. The synergic interactions of π-π-interaction of perylene and Azo units and van der Waals force are contributed to the formation of fibers [29], but the hydrogen bond between PBI units was weakened by DMF. As the alkyl chain length increases, the van der Waals force increases and the alkyl chains may entangle with each other. **PBI2** formed short fibers and **PBI3** just formed fiber-like assemblies, respectively. Subsequently, the above aggregates were irradiated with 365 nm wavelength light for 30 min to ensure the complete transformation of *trans*- to *cis*-configuration of Azo groups. For **PBI1**, the morphology of assembly remained fibers but the width of them changed from 90 ± 2 nm to 60 ± 5 nm. For **PBI2**, the length of fibers became shorter and their form a slender willow leaf-like structure. For **PBI3**, the morphology changed from fiber-like shape to nanoparticles. Subsequently, we irradiated these aggregates with 436 nm wavelength light for 30 min to ensure the Azo groups to transform from *cis*- to *trans*-configuration thoroughly. The morphology of **PBIs 1–3** aggregates all showed the recovering tendency. For **PBI1**, the assembly remained fibers and the width of them increased to 70 ± 3 nm. For **PBI2**, the morphology retransformed to the fibers but with a much narrow width. For **PBI3**, the assembly returned to a fiber-like shape but with a much bigger size. It showed that the morphology of assembly can be regulated by reversible photoisomerization of Azo groups, especially for **PBI2**.

## 4. Conclusions

In summary, we designed and synthesized three Azo-functionalized PBI derivatives with different alkyl chain lengths. They were able to assembly to supramolecular structures by the cooperation of π-π stacking, hydrogen bond, and the van der Waals force. The Azo groups in **PBIs 1–3** were able to take photoisomerization and the magnitude of intermolecular interaction force is the key factor for the photoisomerization process. The TEM test showed that the longer alkyl chain was not conducive to the formation of the fibers. In DMF, **PBI1** and **PBI2** with one and six carbon atom alkyl chains assembled to form the fibers, while **PBI3** with 12 carbon atom alkyl chains could only form the fiber-like assembly. For perylene bisimide derivative **PBI2** with a medium alkyl chain length, when Azo groups were in *trans*-configuration, the short fibers formed. By 365 nm ultraviolet light irradiation, the assembly transformed into a slender willow leaf-like structure due to the transformation of *trans*- to *cis*-configuration of Azo groups. When the solution was irradiated with 436 nm visible light, the Azo groups transformed from *cis*- to *trans*-configuration, which resulted in re-transformation to the fibers. By reversible *trans-cis* photoisomerization, the morphology of assembly can be regulated. The current results will provide a way for designing light-responsive PBI-based supramolecular polymers.

## Figures and Tables

**Table 1:** Photoisomerization rate of PBIs in DMF.

Entry	*K*_e_/s^−1^	*K*_h_/s^−1^
**PBI1**	0.0767	0.0257
**PBI2**	0.0955	0.0262
**PBI3**	0.0896	0.0269

## Supplementary Materials

The detailed synthesis of compounds a–d are available online at https://www.mdpi.com/2073-4360/11/7/1143/s1.
