# Supplementary material for: Synthesis and Photocontrolled Supramolecular Self-Assembly of Azobenzene-Functionalized Perylene Bisimide Derivatives
Source: Polymers (Basel). 2019 Jul 3;11(7):1143. doi: 10.3390/polym11071143 (PMC6681406; doi:10.3390/polym11071143)
Supplement: Supplementary file 1 [file polymers-11-01143-s001.pdf]

Supporting Information for

Synthesis and Photocontrolled Supramolecular Self-assembly of Azobenzene-functionalized Perylene Bisimide Derivatives

Weikang Ling, Xiaoxiao Cheng, Tengfei Miao, Shuangshuang Zhang, Wei Zhang* and Xiulin Zhu

Experiment part

1. Synthesis

1.1 Synthesis of 1a, 2a and 3a.

p-Methoxyaniline (6.16 g, 50 mmol), deionized water (40 mL) and concentrated hydrochloric acid (15 mL) were added to a 250 mL round flask. The solution was kept at 0 ^{O}C for 30 min with vigorous mechanical agitation. A solution of sodium nitrite (3.50 g, 100 mmol) in deionized water (40 mL) was slowly added to the mixture. The solution was kept at 0 ^{O}C for another 30 min with vigorous mechanical agitation to produce the diazonium salt solution. Then added dropwise to a solution of phenol (8.00 g, 85 mmol), sodium hydroxide (4.00 g, 100 mmol), and sodium bicarbonate (4.20 g, 50 mmol) in a 500 mL glass bottle. The solution was kept at 0 ^{O}C for 3 h under mechanical agitation. After the reaction, the mixed solution was filtered and washed with deionized water. The crude product was recrystallized from ethyl alcohol and dried in vacuum to get 10.53 g of a yellow solid. The compounds 2a and 3a were synthesized via the similar procedures.

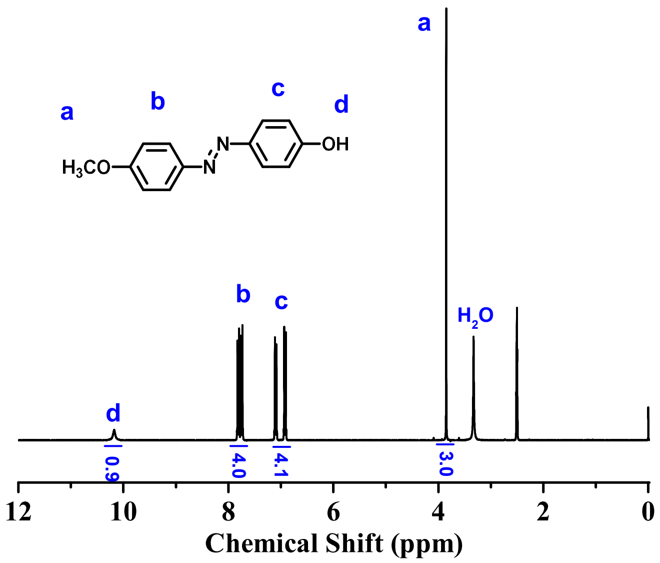

Figure S1. ^{1}H NMR spectra of **compound 1a** in DMSO-d_6.

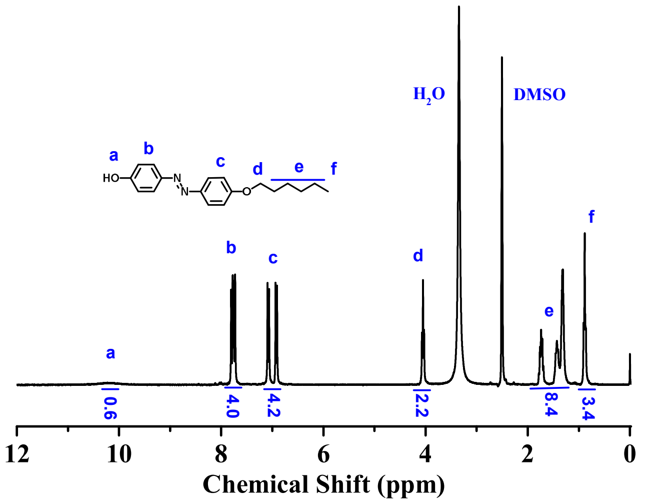

Figure S2. ^{1}H NMR spectra of **compound 2a** in DMSO-d_6.

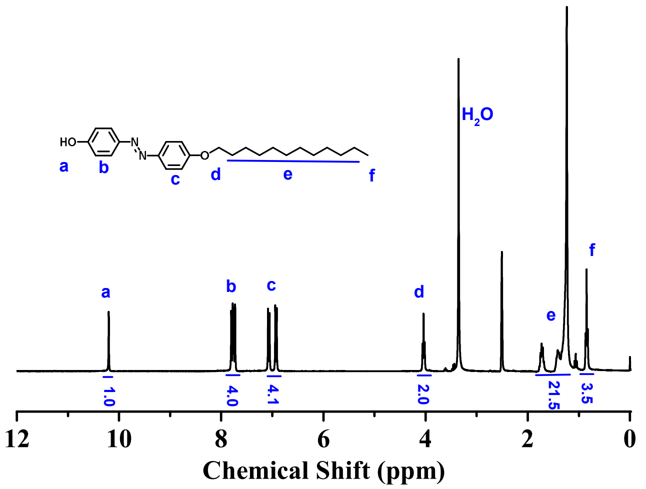

Figure S3. ^{1}H NMR spectra of **compound 3a** in DMSO-d_6.

1.2 Synthesis of 1b, 2b and 3b.

Compound 1a (2.97 g, 13 mmol),1,6-dibromohexane (6.30 g, 26 mmol), potassium carbonate (2.76 g, 20 mmol) and potassium iodide (0.50 g, 3 mmol) were added to acetone (80 mL) under magnetic stirring, and the reaction mixture was heated to 50 °C and stirred for 10 h. After the reaction, the mixture was poured into deionized water and filtered. The crude product was dissolved in ethyl acetate by heating and the hot solution was immediately filtered to remove the inorganic residue. Then the product was dried under vacuum to get 2.92 g of yellow solid. The

compounds 2b and 3b were synthesized via the similar procedures.

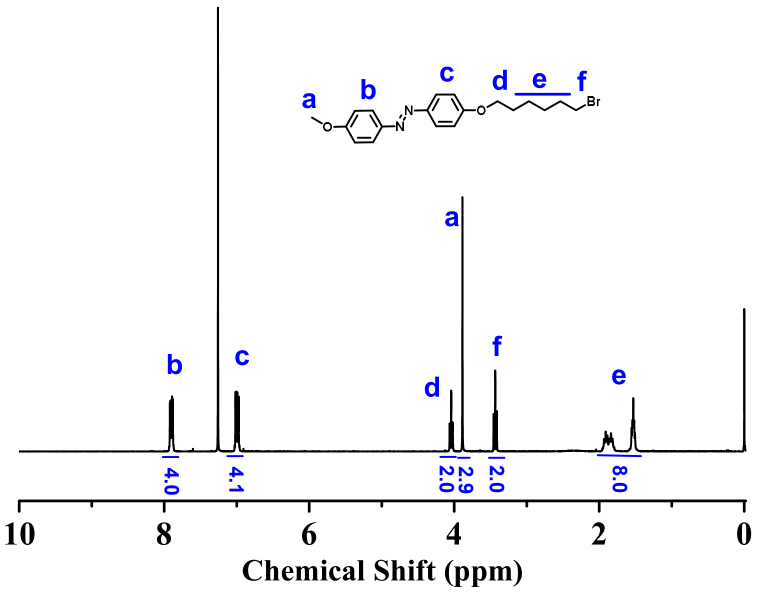

Figure S4. ^{1}H NMR spectra of **compound 1b** in $CDCl_3$.

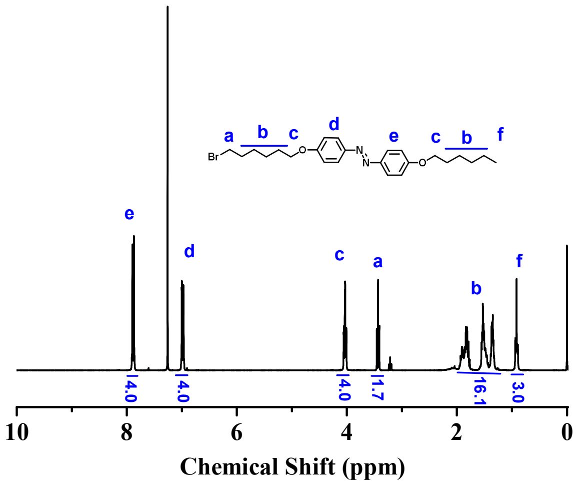

Figure S5. ^{1}H NMR spectra of **compound 2b** in $CDCl_3$.

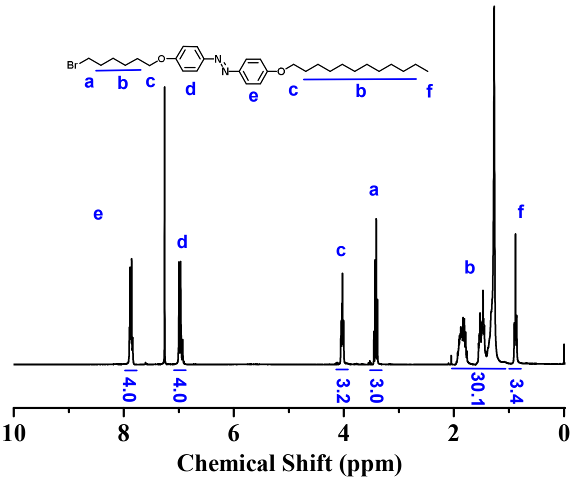

Figure S6. ^{1}H NMR spectra of **compound 3b** in $CDCl_3$.

1.3 Synthesis of 1c, 2c and 3c.

The alkyloxylation reaction was carried out by refluxing compound 1b (2.34 g, 6 mmol), methyl gallate (0.30 g, 1.65 mmol), potassium carbonate (0.83 g, 6 mmol) and potassium iodide (0.14 g, 0.9 mmol) in 2-butanone (50 mL), and the reaction mixture was stirred at 75 °C for 48 h. After the reaction, the solution was poured into dilute HCl solution. The yellow precipitate was filtered and washed with water until the liquid phase became neutral. The product was dried in vacuum. The crude product was recrystallized from $CHCl_3$, then dried under vacuum to get 1.63 g of a yellow solid. The compounds 2c and 3c were synthesized via the similar procedures.

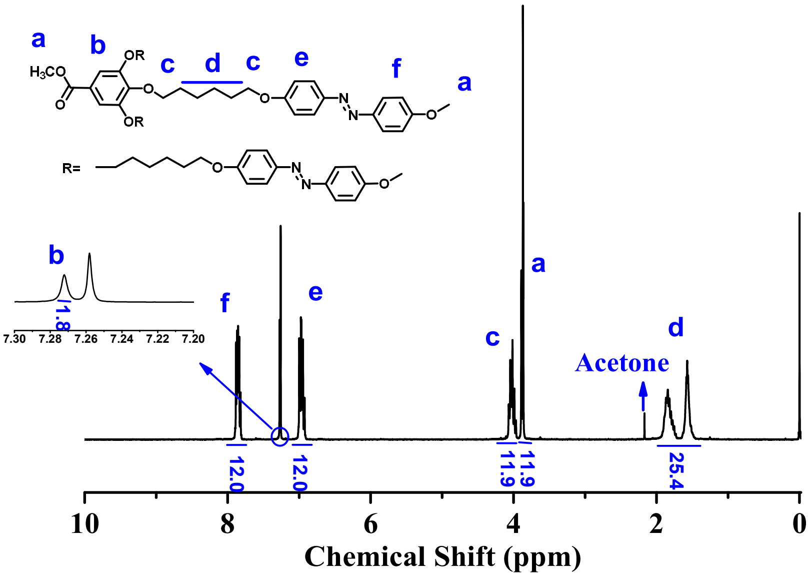

Figure S7. ^{1}H NMR spectra of **compound 1c** in $CDCl_3$.

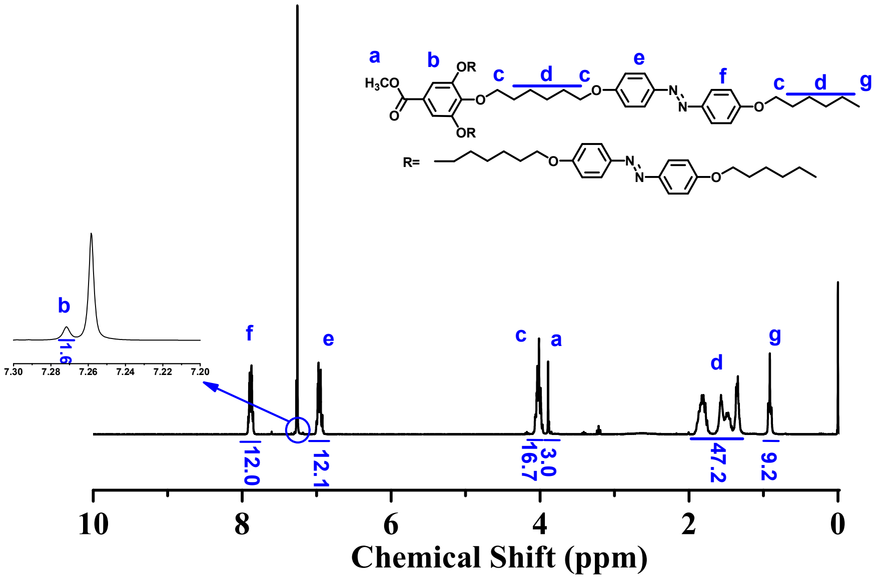

Figure S8. ^{1}H NMR spectra of **compound 2c** in $CDCl_3$.

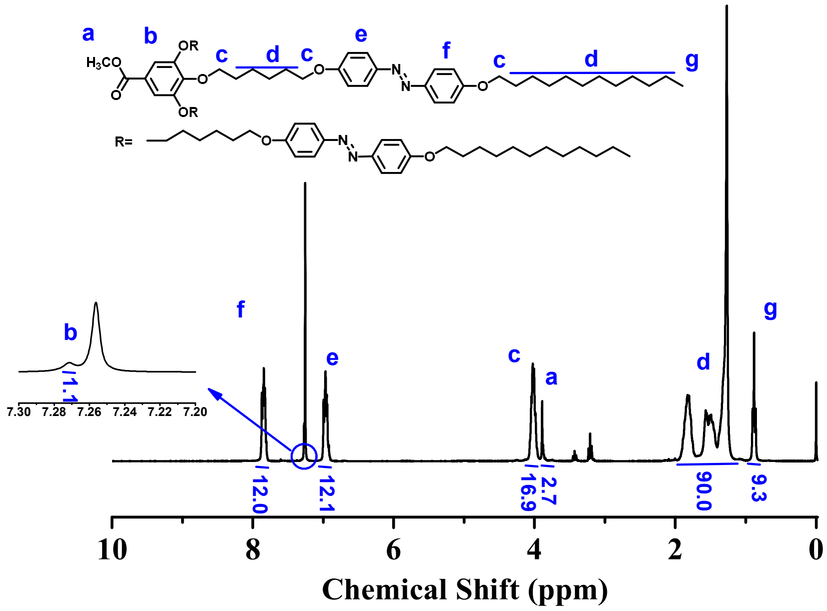

Figure S9. ^{1}H NMR spectra of **compound 3c** in $CDCl_3$.

1.4 Synthesis of 1d, 2d and 3d.

The solution (50 mL) of sodium hydroxide (4.37 g, 110 mmol) was added to the THF solution (50 mL) of compound 1c (4.83 g, 3.65 mmol), and few drops of CH_3OH were simultaneously added. The mixture were stirred and refluxed for 16 h at 85 °C. After the reaction, the reaction mixture was cooled to room temperature and acidified to pH < 7 with HCl. The yellow precipitate was filtered and washed with water until the liquid phase became neutral. The product was dried in vacuum. The crude product was recrystallized from $CHCl_3$ and dried under vacuum to get 3.82 g of a

yellow solid. The compounds 2d and 3d were synthesized via the similar procedures.

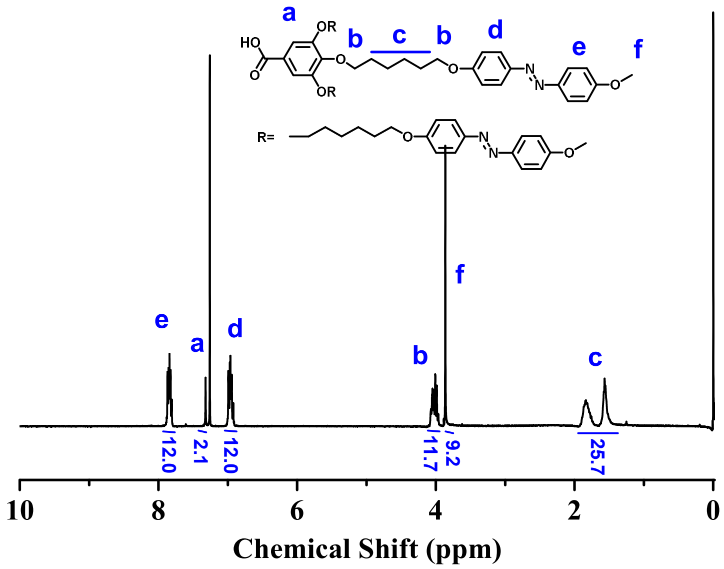

Figure S10. ^{1}H NMR spectra of **compound 1d** in $CDCl_3$.

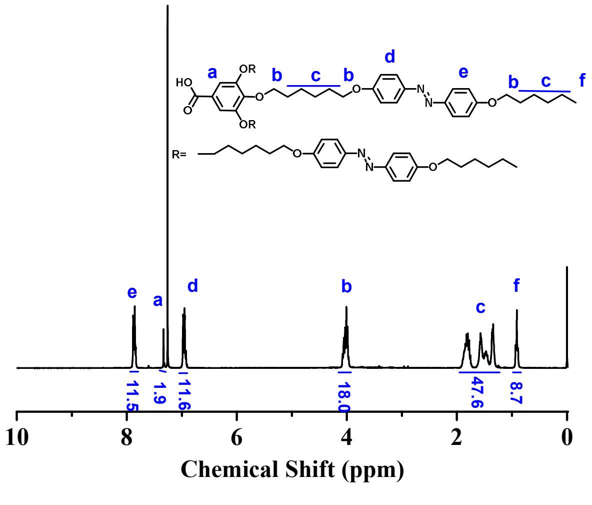

Figure S11. ^{1}H NMR spectra of **compound 2d** in $CDCl_3$.

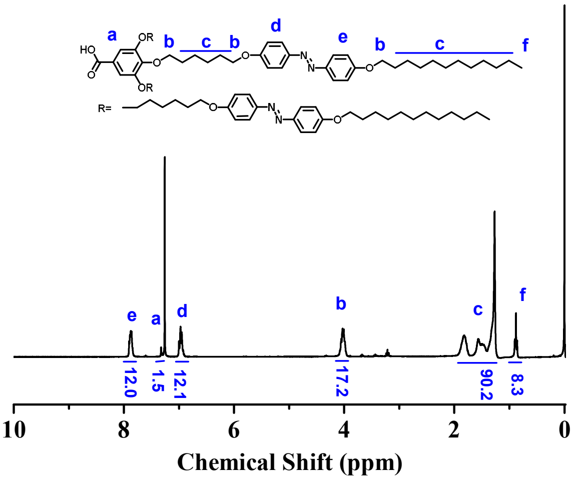

Figure S12. ^{1}H NMR spectra of **compound 3d** in $CDCl_3$.

1.5 ^{1}H NMR spectra of compounds 1e, 2e and 3e

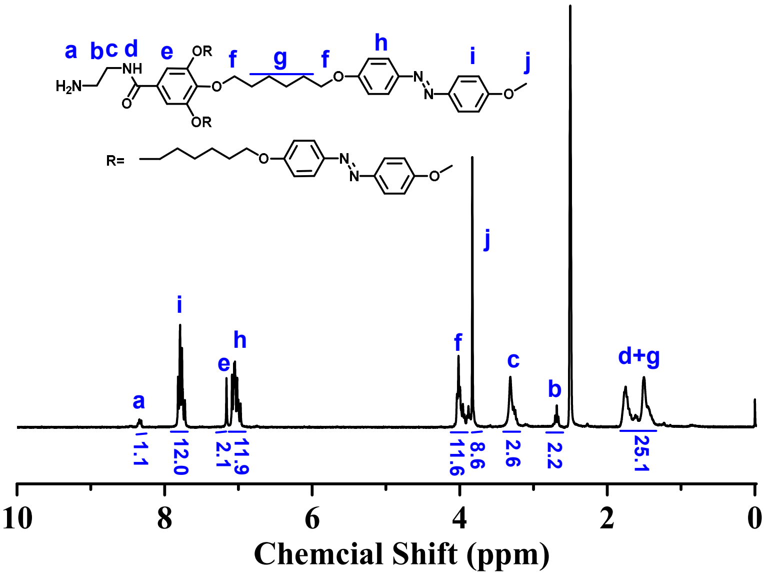

Figure S13. ^{1}H NMR spectra of **compound 1e** in DMSO-d_6.

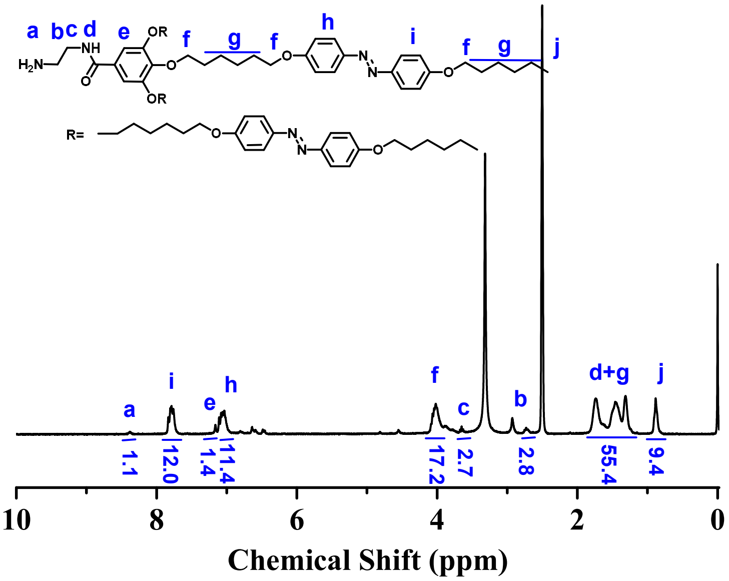

Figure S14. ^{1}H NMR spectra of **compound 2e** in DMSO-d_6.

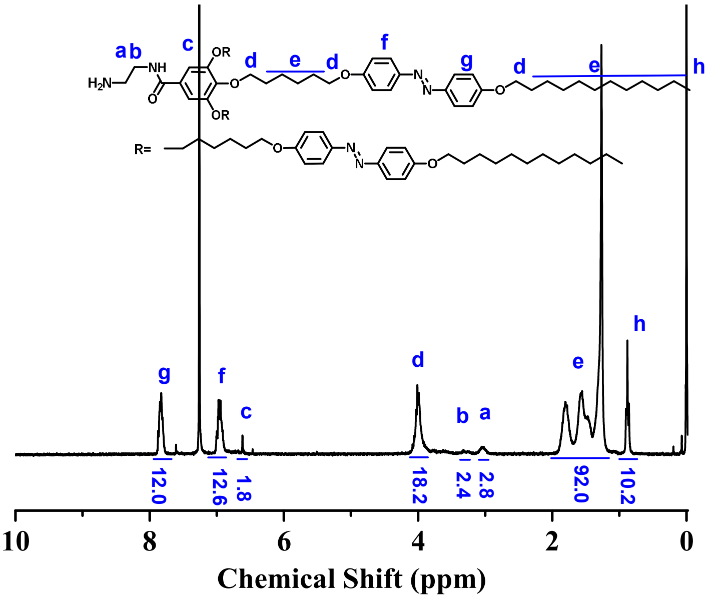

Figure S15. ^{1}H NMR spectra of **compound 3e** in $CDCl_3$.

1.6 ^{1}H NMR spectra of PBIs 1-3.

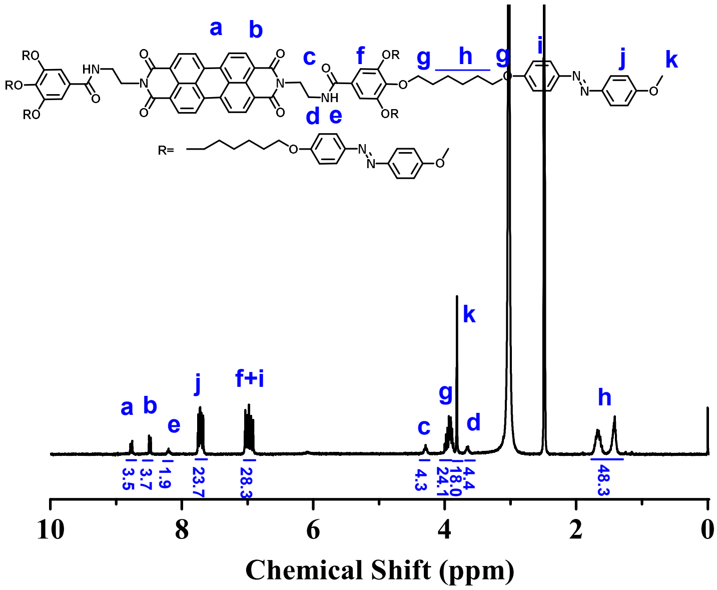

Figure S16. ^{1}H NMR spectra of **PBI1** in DMSO-d_6.

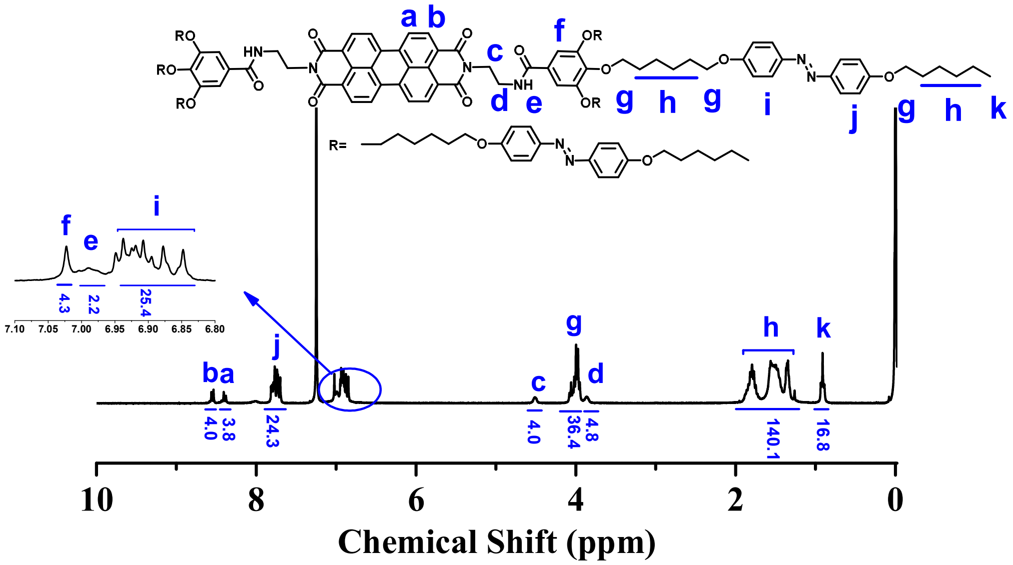

Figure S17. ^{1}H NMR spectra of **PBI2** in $CDCl_3$.

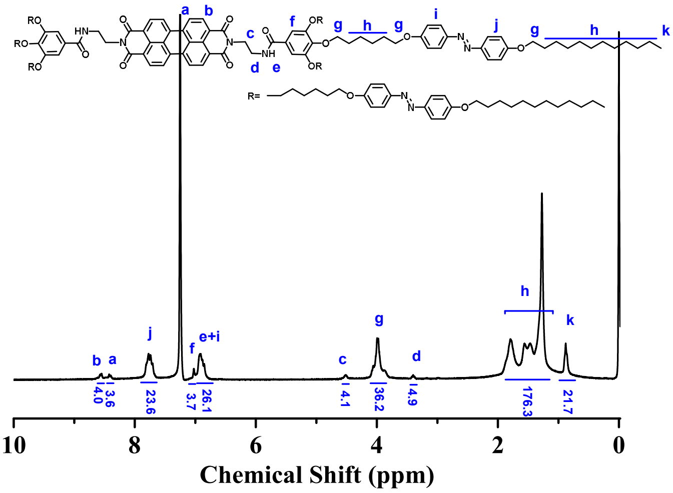

Figure S18. ^{1}H NMR spectra of **PBI3** in $CDCl_3$.

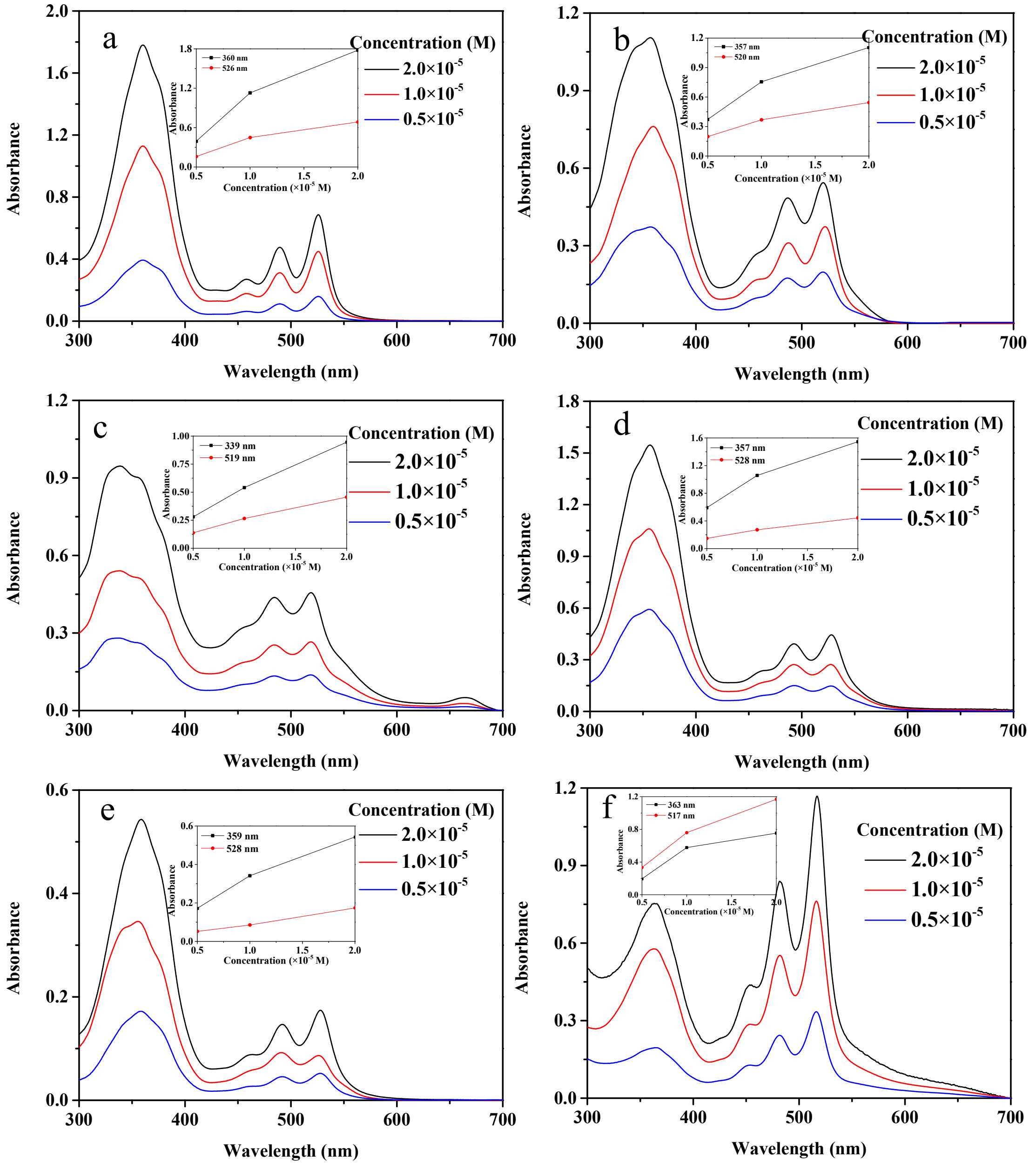

Figure S19. UV-vis spectra of **PBIs 1-3** with different concentrations in DMF (a-c for **PBIs 1-3**) and DCE (d-f for **PBIs 1-3**). The inserted image is the dependence of UV-vis absorption intensity with PBI concentration.

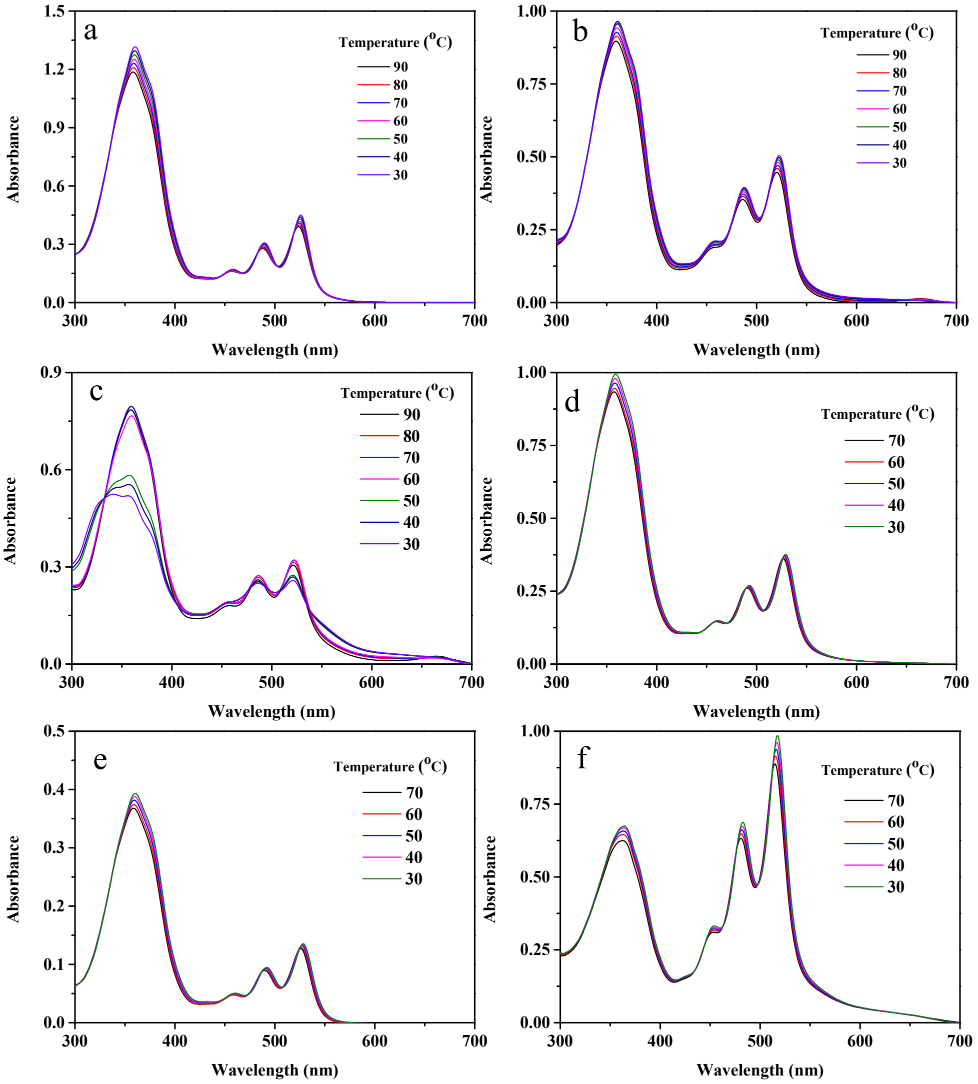

Figure S20. UV-vis spectra of **PBIs 1-3** at different temperature in DMF (a-c for **PBIs 1-3**) and DCE (d-f for **PBIs 1-3**).
